# Computational Insights Into Smart Bioelectronics in Digital Health Care (2020-2024): Topic Modeling Study

**DOI:** 10.2196/83092

**Published:** 2026-06-23

**Authors:** JiWon Bae, JiHoon Lee, Pildong Hwang, Ji Eun Shin, Sung Ryul Shim, Jong-Yeup Kim, Seunghee Lee

**Affiliations:** 1 Konyang Medical Data Research Group-KYMERA, Konyang University Hospital, 158 Gwanjeodong-ro, Seo-gu, Daejeon, Republic of Korea, 82 426008679; 2 Department of Biomedical Informatics, College of Medicine, Konyang University, Daejeon, Republic of Korea; 3 Department of Otorhinolaryngology-Head and Neck Surgery, College of Medicine, Konyang University Hospital, Daejeon, Republic of Korea

**Keywords:** smart bioelectronics, keyword analysis, topic modeling, research trends, PubMed

## Abstract

**Background:**

Smart bioelectronics are electronic medical devices that combine hardware and artificial intelligence (AI)–based software. These convergent medical devices analyze bio-signals measured through hardware using AI algorithms and deliver physical stimulation to enhance therapeutic effects.

**Objective:**

This study aimed to systematically analyze recent research trends in smart bioelectronics to understand their evolving role in digital health care and to provide evidence-based insights for shaping future research and development strategies.

**Methods:**

A total of 92 publications indexed in PubMed between 2020 and 2024 were analyzed. Latent Dirichlet allocation–based topic modeling, optimized using coherence scores, was applied to identify latent research themes.

**Results:**

The results indicate a steady increase in related research over the past 5 years, along with a clear shift in research focus from bio-signal sensing and bioelectronic device materials toward AI-driven analysis and disease-oriented applications, ultimately evolving into intelligent and adaptive bioelectronic therapeutic systems. Three major research topics were identified: bio-signal–based neuromodulation (n=23, 25%), AI-driven neurological disease analysis (n=32, 34.7%), and implantable bioelectronics and biomaterials (n=37, 40.2%).

**Conclusions:**

By mapping the evolving landscape of smart bioelectronics, this study provides valuable insights into their multidisciplinary development and highlights their potential applications in clinical decision support, personalized rehabilitation, and next-generation medical device innovation.

## Introduction

### Background

Bioelectronics are medical devices that provide therapeutic effects through physical stimulation without using drugs and are receiving attention in the medical community because they can solve the side effects and misuse problems associated with existing drug treatments [1]. Although existing drug treatments use chemical components to cause biochemical reactions in the body to relieve symptoms, bioelectronics directly stimulate nerves to induce therapeutic effects; therefore, their mechanism of action is clear, and side effects are relatively low. In addition, real-time treatment monitoring and immediate feedback are possible, allowing for more precise treatment.

In the past, bioelectronics were used in a limited manner by inserting them into the body, such as pacemakers, but recently, as they have evolved into noninvasive methods, an environment is being created where patients can receive treatment at home [2]. In addition, the market is rapidly expanding as clinical efficacy has been proven for various diseases such as depression, insomnia, headache, dementia, and epilepsy. In the global market, commercialization of bioelectronics is actively progressing, centered around the United States and Europe, and the number of related products is gradually increasing. In contrast, the domestic bioelectronics market is still in its initial stages, and product development and clinical application are not active, so competitiveness is low. Accordingly, research and new technology development for effective clinical application of bioelectronics are essential in Korea as well, and it is necessary to increase treatment precision and strengthen the competitiveness of the medical device industry through the combination with artificial intelligence (AI), big data, and wearable technology.

In the health care field, the amount of data, such as research papers, patents, and clinical data, is increasing exponentially. These data provide important research insights, but the volume is so large that manual analysis is limited. However, data analysis is essential for suggesting research directions and exploring innovative treatments. Accordingly, research trend analysis using text mining and topic modeling is actively being conducted.

Text mining is a technology that extracts meaningful information by formalizing unstructured data and is used to quantitatively understand research trends by analyzing research papers and patent documents. Topic modeling is a technique that automatically derives key topics from large numbers of documents and is useful for classifying topics in research papers and analyzing research trends [3]. Through these approaches, it is possible to analyze which topics are currently being studied most actively and how the proportion of specific studies has changed over time. A study that analyzed the main topics of health literacy–related research identified research trends over the past 10 years and suggested future research directions [4]. In another study, the topic modeling techniques were applied to derive key technologies and major issues in the analysis of domestic research trends related to the Fourth Industrial Revolution [5]. Using these techniques can contribute to a more systematic understanding of the latest trends in electronic pharmaceutical research and help establish future research directions.

**Figure 1.**
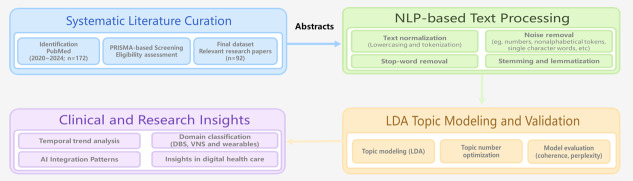
Analytical framework for smart bioelectronics research trend analysis: the workflow began with systematic literature curation using a PRISMA (Preferred Reporting Items for Systematic Reviews and Meta-Analyses)–based screening process, followed by natural language processing (NLP)–based text preprocessing, including normalization, tokenization, and stop-word removal. Latent Dirichlet allocation (LDA) was applied for topic modeling, with model evaluation and topic number optimization performed using coherence metrics. The resulting topics were further analyzed to identify temporal research trends, domain-specific classifications, and emerging artificial intelligence (AI) integration patterns, providing clinically relevant and translational insights in digital health care. DBS: deep brain stimulation: VNS: vagus nerve stimulation.

This study aims to analyze the trends in smart electronic drug research over the past 5 years, to analyze how research on the topic of “smart electronic drugs” is progressing, and to suggest future research directions. Relevant studies were identified from the PubMed database using bioelectronics-related keywords combined with AI-related terms in the titles or abstracts. Frequency analysis and latent Dirichlet allocation (LDA)–based topic modeling were applied. First, we analyzed the quantitative research flow and meaning by identifying the trends and changes in the core keywords of smart electronic drugs research over time through frequency analysis. Next, we analyzed the main topics and distribution of the research content using LDA-based topic modeling to explore overall research trends and their implications (Figure 1).

In addition, this study set 3 research topics to identify trends in smart bioelectronics research and suggest future directions, and the following implications were derived from the analysis results of each topic:

- Research topic 1: how have research trends in smart bioelectronics changed over the past 5 years, and what do they mean?
- Research topic 2: what are the core keywords and main topics of smart bioelectronics research trends, and what do they mean?
- Research topic 3: what are the characteristics of the main topics derived from the topic analysis, and what strategic directions can be derived from each topic?

### Smart Bioelectronics

Smart bioelectronics are advanced technologies that combine AI-based software with hardware-based bio-stimulation devices to measure bio-signals in real time, analyze them with AI algorithms, and provide physical stimulation optimized for an individual’s condition, thereby maximizing therapeutic effects [6]. Unlike conventional chemical drug treatments, smart bioelectronics directly stimulate nerves and tissues to induce physiological responses, enabling precise and personalized treatment. Consequently, they are attracting attention as a new alternative for chronic diseases, neurological diseases, and rehabilitation treatment.

Meanwhile, the term referring to electronic drugs first appeared in earnest in 2013 when GlaxoSmithKline introduced a therapeutic device that controls neural circuits through electrical stimulation and used the expression “electroceutical.” However, the terminology in this field has not been unified to date, and the terms “electroceutical” and “bioelectronic medicine” are used interchangeably, and “bioelectronics” and “electronic drugs” are also used in various ways depending on the literature. As this confusion of terms can obscure technical and academic identities, it is necessary to establish clear concepts and a classification system in future research and industrialization stages.

### Topic Modeling

Topic modeling is a technique that uses machine learning and natural language processing technology to analyze large amounts of text data and automatically extract major topics from documents [3]. It is used to understand the content of documents, group related topics, and explore hidden patterns within data. Topic modeling is particularly useful for extracting meaningful information from massive document datasets, such as research papers, news articles, and electronic medical records, and is widely applied across various fields, including medicine and biology.

Recently, topic modeling has been increasingly applied in the medical field to analyze large-scale biomedical data and uncover hidden patterns that provide new insights. For example, previous studies have applied topic modeling techniques to analyze public responses to the COVID-19 vaccine using social media data and identified key discussion themes such as vaccine side effects, vaccine hesitancy, and infectious disease management policies [7]. In addition, advanced topic modeling approaches such as the graph attention-embedded topic model have been applied to electronic health record data to identify potential disease-related topics and improve the interpretability of large-scale clinical datasets [8].

Topic modeling is also widely used to identify research trends in scientific literature. For example, previous studies have applied LDA-based topic modeling to analyze research trends in precision medicine and medical device usability by examining large collections of journal papers and media data, enabling the discovery of emerging research themes and key industry elements [9,10]. In addition, studies by Özyurt et al [11-14] demonstrated effective approaches for reporting LDA parameters, selecting optimal topic numbers based on coherence scores, and interpreting temporal research trends. These studies provide important methodological benchmarks for conducting bibliometric topic modeling analysis.

Following these approaches, this study applies LDA-based topic modeling to analyze research trends in smart bioelectronics literature.

## Methods

### Data Collection

This study used the PubMed database to collect publications related to smart bioelectronics. PubMed is a widely used biomedical database that provides access to references and abstracts in the life sciences and biomedical fields, primarily including MEDLINE-indexed literature.

The literature search targeted studies related to bioelectronics and electroceutical research combined with AI. Because there is currently no dedicated Medical Subject Headings term for electroceutical or bioelectronic medicine, the search was conducted using keywords appearing in the titles and abstracts of papers.

To capture the heterogeneous terminology used in this emerging research area, 2 complementary PubMed database search strategies were used. The first query focused on electroceutical and neuromodulation-related terminology combined with AI-related keywords, while the second query targeted bioelectronics together with AI, sensor, wearable, and closed-loop control concepts.

The criteria for paper selection were as follows:

- Studies containing bioelectronics-related keywords (eg, “bioelectronics,” “electroceutical,” or “neuromodulation”) and AI-related terms (eg, “artificial intelligence,” “machine learning,” “deep learning,” or “reinforcement learning”) in the title or abstract
- Papers published between January 1, 2020, and December 31, 2024
- Studies involving human subjects
- Papers providing free full text and abstracts
- Exclusion of review papers

The PubMed search was conducted using the following queries:

- Query 1: (electroceutical*[Tiab] OR neuromodulation[Tiab] OR “bioelectronic medicine”[Tiab] OR “vagus nerve stimulation”[Tiab] OR “nerve stimulation”[Tiab] OR “deep brain stimulation”[Tiab]) AND (“artificial intelligence”[Tiab] OR “machine learning”[Tiab] OR “deep learning”[Tiab] OR “reinforcement learning”[Tiab]) AND (“2020/01/01”[pdat] : “2024/12/31”[pdat]) AND humans[MeSH Terms] AND fha[Filter] NOT review[pt]
- Query 2: (bioelectronic*[Tiab] OR electroceutical*[Tiab] OR “bioelectronic medicine”[Tiab]) AND (“Artificial Intelligence”[Tiab] OR AI[Tiab] OR “Machine Learning”[Tiab] OR “Deep Learning”[Tiab] OR sensor*[Tiab] OR wearable*[Tiab] OR “closed-loop”[Tiab] OR “closed loop”[Tiab]) AND (“2020/01/01”[pdat] : “2024/12/31”[pdat]) AND humans[MeSH Terms] AND fha[Filter] NOT review[pt]

After applying the inclusion criteria and conducting manual screening based on titles and abstracts, 49 studies from query 1 and 43 studies from query 2 were retained. Duplicate records were checked using title and DOI matching; however, no duplicate studies were identified between the 2 query results. Consequently, a total of 92 studies were included in the final dataset for the subsequent text mining and LDA-based topic modeling analysis, as listed in Multimedia Appendix 1 [15-106].

### Ethical Considerations

This study used publicly available bibliographic data retrieved from PubMed. No individual participant data or identifiable personal information was included. Therefore, institutional review board approval and informed consent were not required for this study*.*

### Data Preprocessing

Preprocessing was performed to ensure the quality of the bibliographic data from the 92 selected papers. The preprocessing steps are as follows. First, the entire text was converted to lowercase to ensure consistency in word notation. To select only the words essential for semantic analysis, 7571 nouns were selected through headword extraction and part-of-speech tagging. Subsequently, special characters and stop words were removed, as they were unnecessary for analysis. Stop-word removal was performed using the Natural Language Toolkit (NLTK) default stop-word dictionary [12] to remove common stop words. Additionally, 2886 stop words were removed, including custom stop words such as “system,” “study,” and “patient,” which frequently appeared in papers but did not contribute to semantic differentiation. Finally, 49 single-letter words were filtered out, resulting in 4452 text data items suitable for analysis (Table 1).

**Table 1.** Data preprocessing steps and main tasks.

Preprocessing stage	Main tasks
Convert to lowercase	Ensuring text consistency
Noun and headword extraction	Perform lemmatization and extract nouns
Remove special characters	Remove unnecessary symbols such as !, ", #, $, %, &, ’, (), and *
Remove stop words	Natural Language Toolkit stop words and custom stop words (eg, “system” and “study”)
Word filtering	Remove 1-letter words

The list of custom stop words was categorized as follows. A total of 122 words with low contribution to semantic distinction were selected and classified into types as follows: (1) research and analysis, (2) technology or system, (3) AI and data, (4) medical or health, (5) sensors and devices, and (6) other terms (Table 2).

**Table 2.** Custom stop-word list.

Category	Stop words
Research and analysis	study, analysis, method, approach, result, performance, accuracy, effect, outcome, score, rate, parameter, process, objective, finding, design, test, validation, conclusion, research, optimization, correlation, scale, mapping, precision, AUC (Area Under the Curve), classifier, classification, regression, crossvalidation, solution, response, beta, reference, application
Technology and systems	system, technology, technique, development, network, tool, array, setting, delivery, management
Artificial intelligence and data	data, model, machine, artificial intelligence, AI (Artificial Intelligence), ML (Machine Learning), SVM (Support Vector Machine), GPT (Generative Pre-trained Transformer), RL (Reinforcement Learning), layer, input, decision, baseline, ratio, difference, set, information, feature, support, forest
Medical and health	patient, participant, subject, cohort, treatment, trial, intervention, condition, risk, brain, motor, activity, efficacy, medication, healthcare, drug, sensation
Sensors and devices	sensor, device, biosensor, bioelectronics, stimulation, DBS (deep brain stimulation), VNS (vagus nerve stimulation), tcVNS (Transcutaneous cervical vagus nerve stimulation), tVNS (Transcutaneous Vagus Nerve Stimulation), STN (Subthalamic Nucleus), neuromodulation
Other	potential, group, area, case, ability, side, number, quality, level, state, time, change, addition, challenge, task, target, background, individual, rating, selection, use, value, field, function, Hz (Hertz), paper, region

### Word Clouds

Word cloud analysis is a representative technique for visualizing text-based unstructured data. It allows intuitive identification of key keywords by varying the font size based on word frequency. In this study, frequency analysis was performed on the collected text data to identify the frequency of key keywords. The preprocessed text was tokenized into words, and keywords were extracted based on their frequency of appearance. Word frequency was calculated using the “explode()” and “value_counts()” functions in the pandas library. To ensure inclusion of all words, the minimum frequency (min_word_count) was set to 5 for extraction. On the basis of these results, visualization was performed using Python’s WordCloud library, allowing an at-a-glance identification of key keywords related to smart bioelectronics.

### LDA Algorithm

LDA was used for topic modeling analysis. It is an unsupervised probabilistic model that represents each document as a mixture of topics and each topic as a distribution over words. The analysis was performed using the LDA algorithm implemented in the Gensim library. The model parameters were set to “iterations=100” and “random_state=4190,” and the number of topics was explored in the range of 3 to 10 with an interval of 1.

The optimal number of topics was determined based on coherence scores, which measure the semantic consistency of keywords within each topic. Perplexity was also examined as a supplementary metric for model evaluation; however, coherence was used as the primary criterion for selecting the optimal number of topics. Furthermore, the results of the LDA models were visualized using pyLDAvis, and the main keywords for each topic were identified based on the top 10 keywords generated by the model.

## Results

### Frequency Analysis Results by Research Period

**Figure 2.**
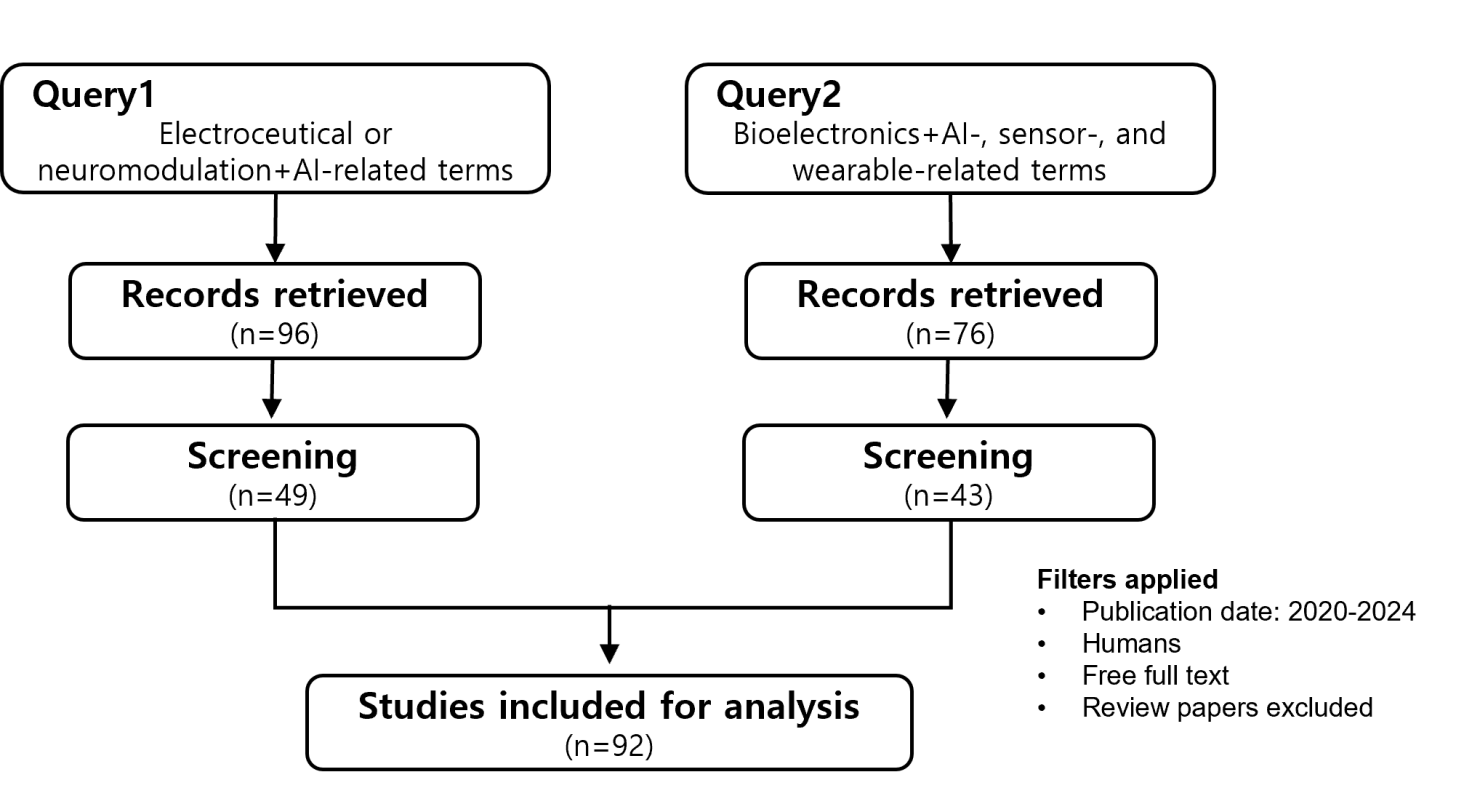
Literature search and dataset construction process. AI: artificial intelligence.

**Figure 3.**
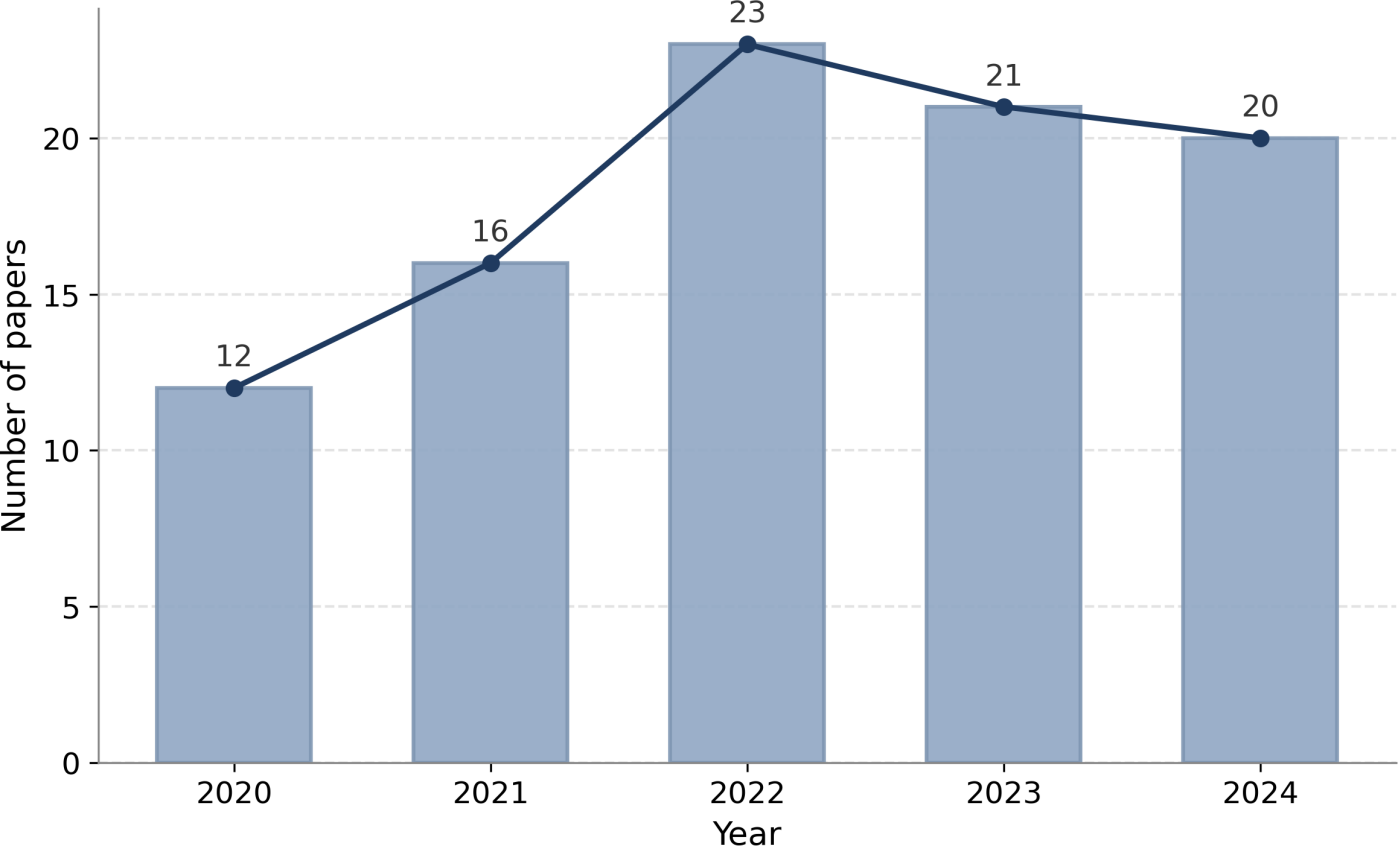
Number of papers by years during the study period (2020-2024). Among the study years, 2022 recorded the highest number of publications, with a total of 23 papers.

The literature search and dataset construction process is illustrated in Figure 2. Because terminology in electroceutical and bioelectronic medicine research remains heterogeneous, some relevant studies may not have been captured by the predefined queries. Nevertheless, the 2 complementary search strategies were designed to improve coverage of this rapidly evolving research field. The annual publication trend for smart bioelectronics-related studies from 2020 to 2024 is shown in Figure 3. The number of publications increased steadily from 12 in 2020 to a peak of 23 in 2022, reflecting the growing research interest in smart bioelectronics (Figure 3).

Using the term frequency–inverse document frequency (TF-IDF) technique, we visualized the most representative keywords for each year from 2020 to 2024 using word clouds. The results reveal a gradual evolution in research focus within the smart bioelectronics field.

In the early stage (2020), keywords such as “biomarkers,” “electrode,” “subcallosal cingulate,” “hydrogel,” “skin,” and “ECG (Electrocardiography)” were dominant, indicating that early research primarily focused on bio-signal sensing technologies and bioelectronic device materials. These keywords reflect the importance of physiological monitoring and electrode-based interfaces in early smart bioelectronics studies. In 2021, disease-oriented terms such as “parkinson disease,” “pain,” and “disease” became more prominent, together with signal acquisition and processing technologies, including “EEG,” “algorithm,” and “control.” This pattern suggests an increasing focus on neurological disorders and the application of computational methods in bioelectronic systems. In 2022, keywords such as “prediction,” “control,” “monitoring,” “therapy,” and “signal” appeared more frequently. These terms indicate the growing integration of computational analysis, signal processing, and control mechanisms in smart bioelectronic systems, reflecting a transition toward data-driven medical device technologies. In 2023, keywords including “algorithm,” “biomarkers,” “sleep,” “tremor,” and “disease” emerged, suggesting increased interest in AI-assisted clinical monitoring and biomarker analysis for neurological and physiological conditions. Finally, in 2024, AI-related keywords such as “learning” and “prediction” became more prominent alongside clinical and device-related terms such as “surgery,” “monitoring,” “tissue,” and “electrode.” These findings indicate a growing integration of AI and predictive analytics in smart bioelectronics research, suggesting a shift toward more intelligent and adaptive bioelectronic therapeutic systems.

### Word Cloud Analysis Results

Word clouds were generated from the top 10 keywords based on the bag-of-words (BoW) and TF-IDF methods. The BoW-based word cloud highlights keywords such as “Parkinson disease,” “biomarkers,” “disease,” “electrode,” “learning,” “prediction,” “algorithm,” “therapy,” “control,” and “EEG.” In contrast, the TF-IDF-based word cloud includes “Parkinson disease,” “biomarkers,” “control,” “prediction,” “electrode,” “EEG,” “disease,” “learning,” “therapy,” and “tissue.”

These keywords can be categorized as follows based on a semantic approach. First, “Sensor/Hardware” refers to terms associated with neural interfaces and bioelectronic sensing components that enable signal acquisition from biological systems, including keywords such as “electrode” and “tissue.” Second, “Application” relates to clinical and therapeutic contexts where bioelectronic technologies are applied for disease management, including terms such as “parkinson disease” and “therapy.” Third, “Analysis/Processing” refers to computational approaches used to analyze and interpret bio-signals, including keywords such as “prediction,” “learning,” “algorithm,” and “control.” Finally, “Signal Monitoring and Biomarkers” refers to the measurement and identification of physiological indicators used to monitor neural activity and disease states, including terms such as “EEG” and “biomarkers.”

“Parkinson disease” emerged as the most prominent keyword in both BoW and TF-IDF analyses. Accordingly, co-occurrence analysis was conducted to examine the relationships between keywords appearing alongside “Parkinson disease.” Network visualization was performed using the top 30 co-occurring keywords.

The results revealed that the research landscape can be broadly categorized into 5 major domains: neural signal acquisition, neuroimaging, clinical symptoms, neuromodulation therapy, and AI-based biomarker analysis. These findings suggest that Parkinson disease research extends beyond clinical observation, encompassing neural signal processing, advanced imaging techniques, therapeutic interventions, and data-driven analytical approaches.

**Figure 4.**
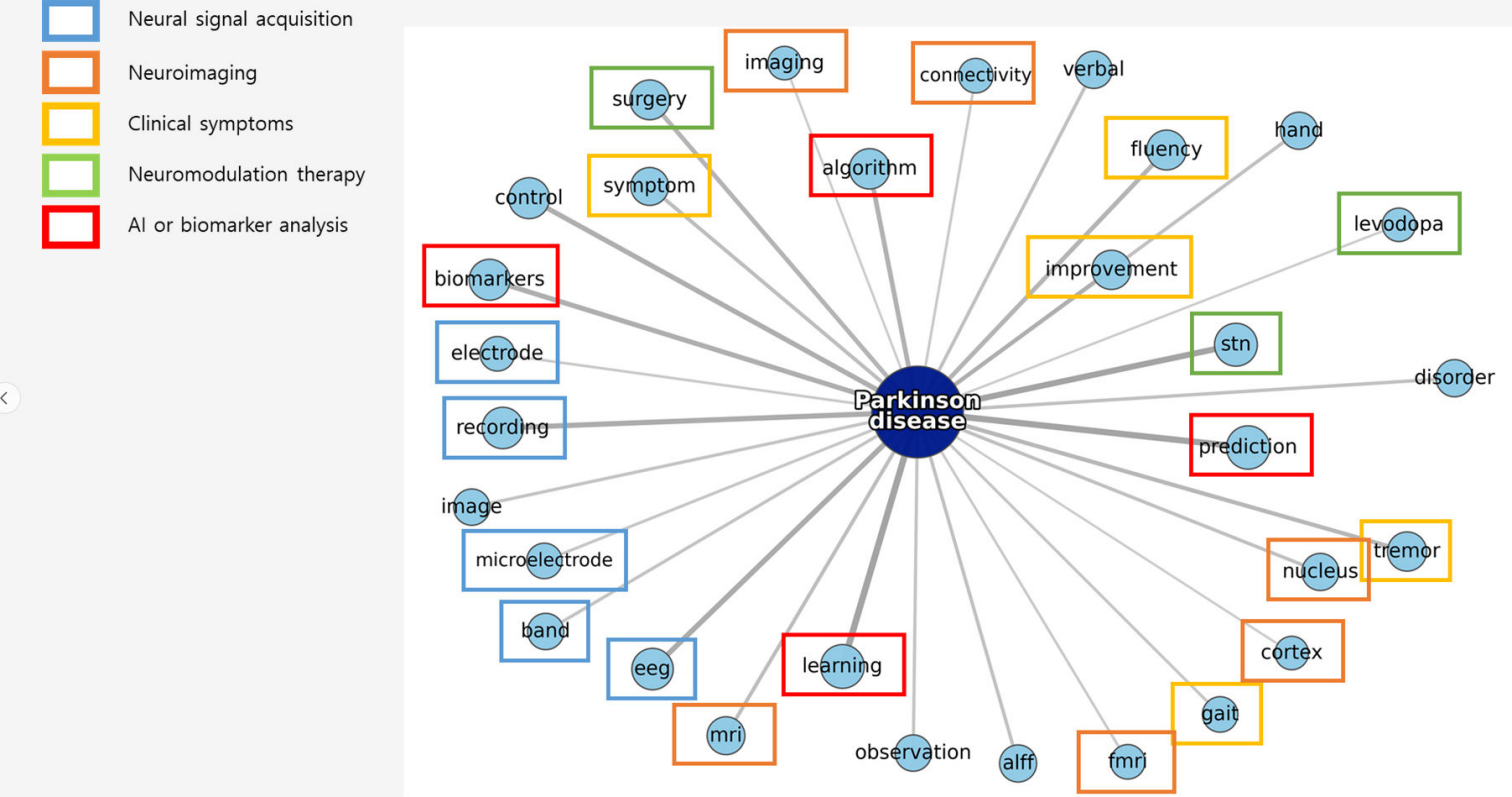
Co-occurrence network for keywords associated with “parkinson disease.” The network was generated based on the top 30 co-occurring keywords. AI: artificial intelligence.

Specifically, neural signal acquisition includes electrocardiogram, electrode, microelectrode, band, and recording; neuroimaging includes magnetic resonance imaging, functional magnetic resonance imaging, imaging, cortex, nucleus, and connectivity; clinical symptoms include tremor, gait, symptom, fluency, and impairment; neuromodulation therapy includes subthalamic nucleus, surgery, and levodopa; and AI-based biomarker analysis includes prediction, algorithm, learning, and biomarkers (Figure 4)

### Topic Modeling Analysis Results

**Figure 5.**
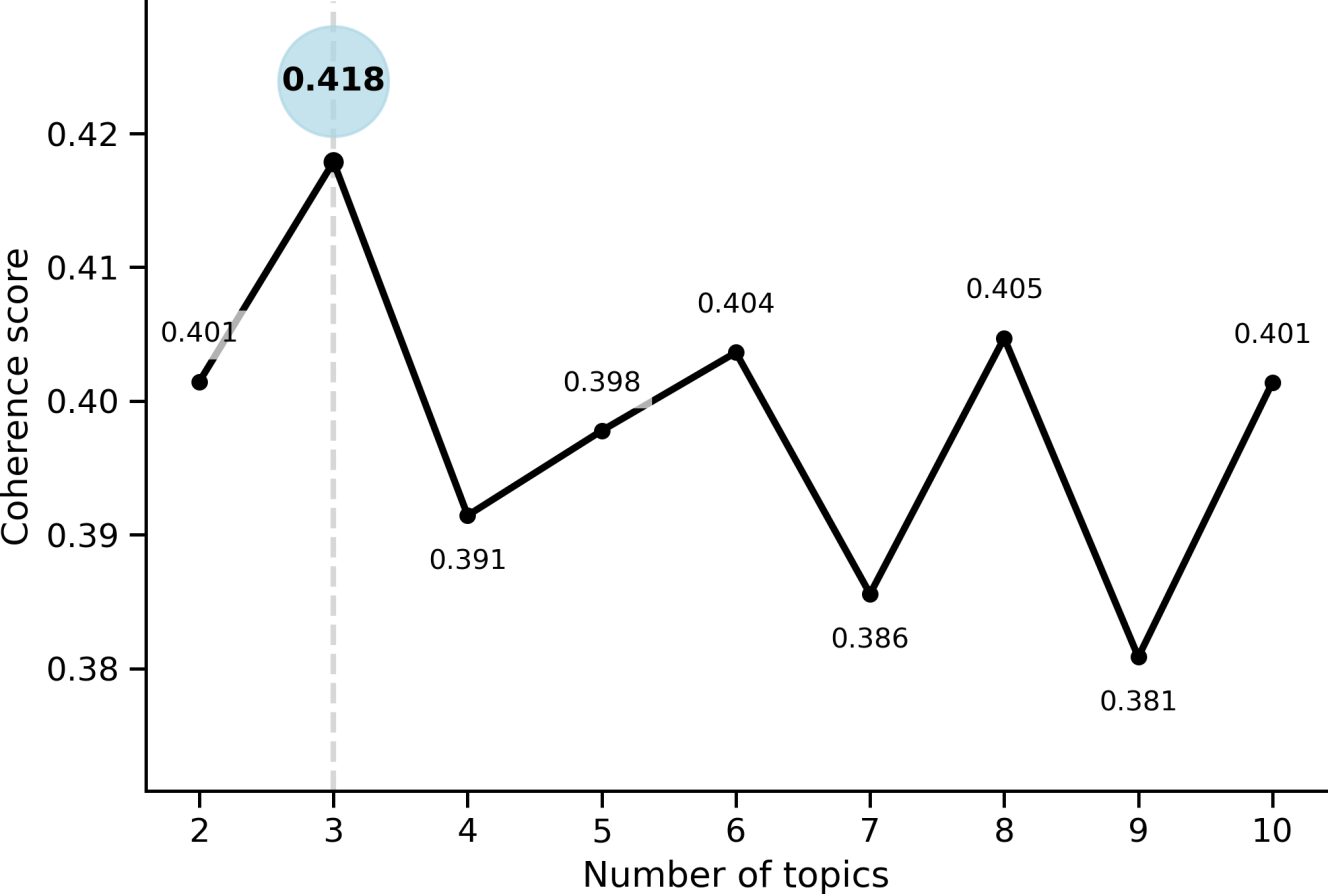
Coherence scores for different numbers of topics.

**Figure 6.**
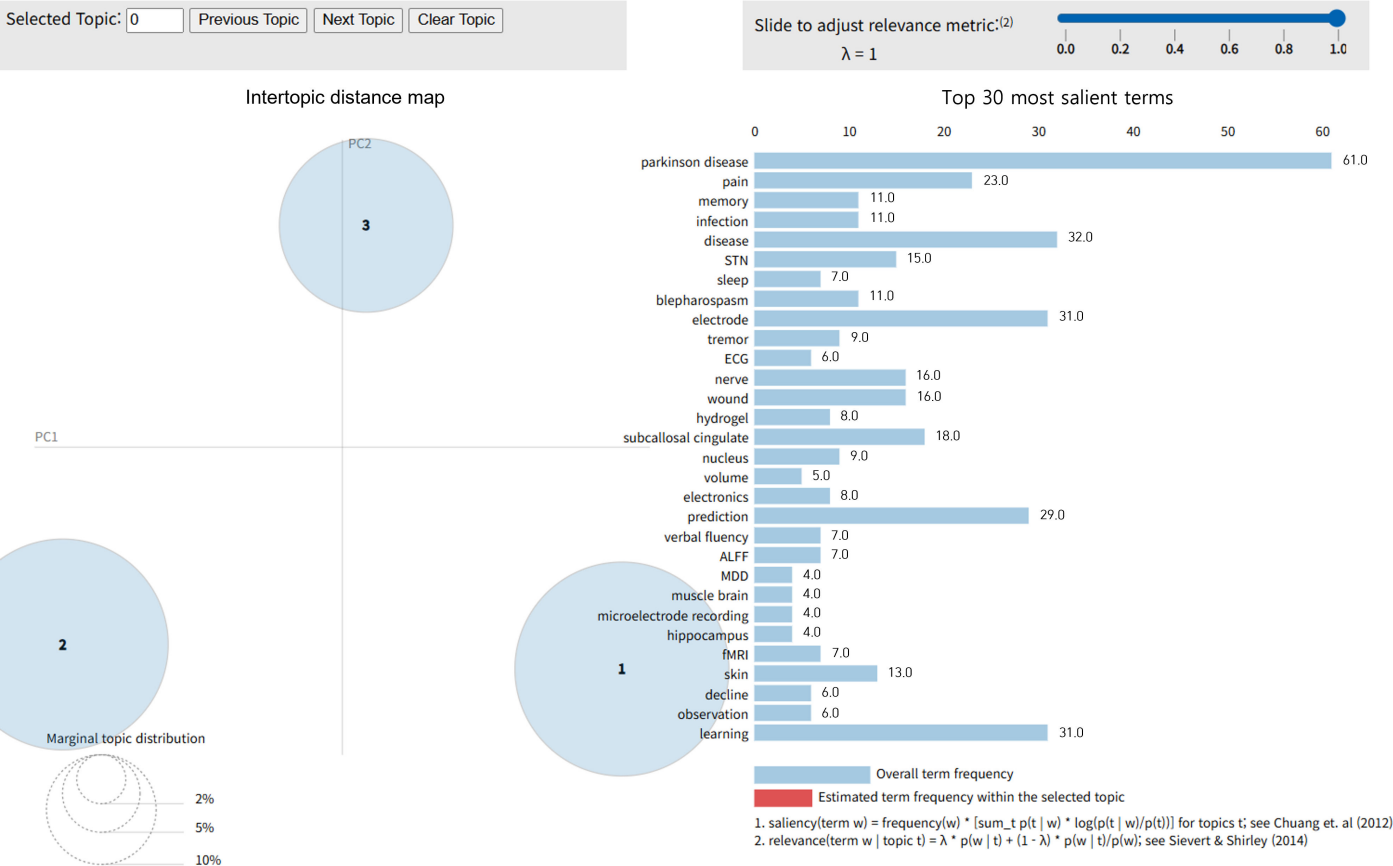
Intertopic distance map visualized using multidimensional scaling. The equations for estimated term frequency within the selected topic were obtained from Chuang et al [107] and Sievert and Shirley [108]. ALFF: amplitude of low-frequency fluctuation; ECG: electrocardiogram; fMRI: functional magnetic resonance imaging; MDD: major depressive disorder; STN: subthalamic nucleus.

The coherence score was used to evaluate the LDA model across different numbers of topics, and the optimal number of topics was determined based on the highest coherence value (Figure 5). Accordingly, 3 topics were selected for the final model, and the intertopic distance map is presented in Figure 6.

Three main thematic areas were identified in smart bioelectronics (Table 3).

**Table 3.** Topic modeling results with representative keywords for each topic.

Topic	Representative keywords
Topic 1: bio-signal–based neuromodulation (publications: 23/92, 25.0%)	pain biomarkers nerve therapy signal tissue monitoring control skin
Topic 2: artificial intelligence–driven neurological disease analysis (publications: 32/92, 34.7%)	parkinson disease disease learning EEG^a^ algorithm control STN^b^ subcallosal cingulate image
Topic 3: implantable bioelectronics and biomaterials (publications: 37/92, 40.2%)	electrode prediction memory infection learning parkinson disease sleep tissue Hydrogel

a EEG: electrocardiogram.

b STN: subthalamic nucleus.

Topic 1 represents bio-signal–based neuromodulation, characterized by keywords such as “pain,” “biomarkers,” “nerve,” and “signal,” indicating a focus on physiological signal acquisition and biomarker-driven therapeutic approaches. From a technical perspective, this topic primarily relies on signal sensing and feature extraction techniques, which form the foundation for subsequent data-driven analysis. Clinically, these approaches enable continuous physiological monitoring and provide objective biomarkers for disease assessment and treatment evaluation.

Topic 2 corresponds to AI-driven neurological disease analysis, including “Parkinson disease,” “EEG,” “learning,” and “algorithm,” suggesting the increasing use of machine learning techniques for the analysis and interpretation of neurological disorders. These approaches are commonly associated with advanced AI methodologies, such as deep learning architectures for bio-signal processing and pattern recognition. From a clinical perspective, these developments contribute to improved diagnostic accuracy, early detection of neurological conditions, and more personalized treatment strategies.

Topic 3 reflects implantable bioelectronics and biomaterials, with representative keywords such as “electrode,” “hydrogel,” “tissue,” and “wound,” highlighting advancements in implantable devices and their interaction with biological systems. The inclusion of terms such as “prediction” and “learning” further indicates the integration of data-driven approaches with implantable systems, enabling intelligent and adaptive therapeutic functions. Clinically, these technologies support real-time monitoring, closed-loop stimulation, and improved treatment efficiency through dynamic adjustment of therapeutic parameters.

Overall, these topics illustrate the evolving research landscape of smart bioelectronics, progressing from bio-signal–based monitoring to AI-driven analysis and, more recently, to intelligent, adaptive, and clinically applicable bioelectronic systems. This progression reflects a paradigm shift toward precision medicine, in which data-driven insights and responsive therapeutic devices are increasingly integrated to address key clinical challenges.

**Figure 7.**
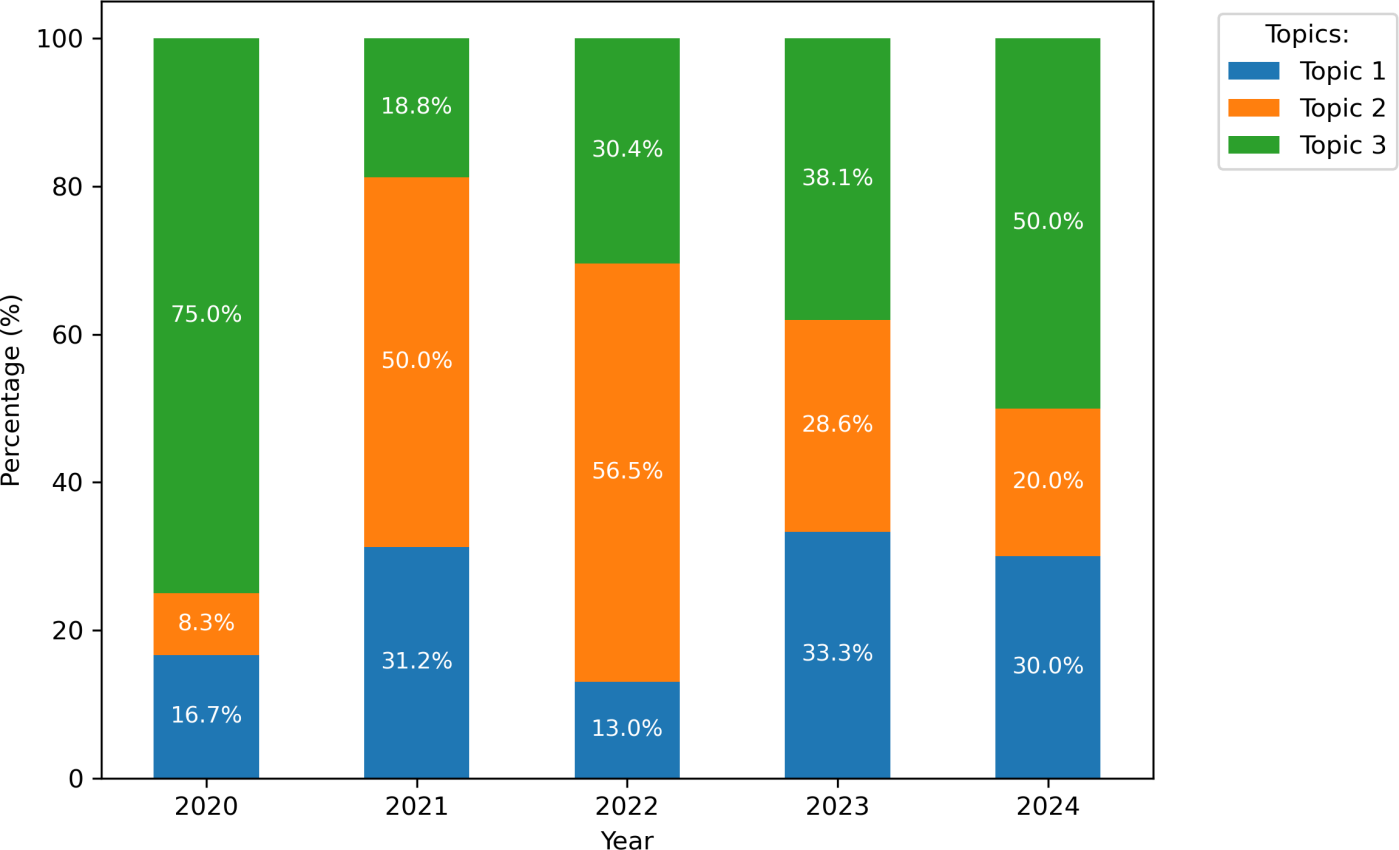
Temporal distribution of latent Dirichlet allocation (LDA)–derived topic proportions from 2020 to 2024.

The temporal distribution of topics reveals a clear evolution in smart bioelectronics research (Figure 7). Early studies were dominated by topic 3, reflecting a strong focus on bioelectronic devices and materials. Between 2021 and 2022, topic 2 became predominant, indicating a rapid expansion of AI-driven analysis and disease-oriented research. In 2023, the distribution became more balanced across topics, suggesting an integration phase [105]. By 2024, topic 3 regained prominence, highlighting a shift toward the practical application and clinical translation of intelligent bioelectronic systems.

Table S1 in Multimedia Appendix 1 summarizes the results of a preliminary review of 92 key papers related to smart bioelectronics [15-106]. These papers were published in leading journals such as *Nat Commun*, *Adv Sci* (*Weinh*), *Sensors* (*Basel*), and *Brain Stimul*. The most frequently appearing keywords were “machine learning,” “bioelectronics,” “deep brain stimulation (DBS),” and “Parkinson’s disease,” indicating that smart bioelectronics research is evolving beyond general AI-based analysis toward clinically oriented applications, particularly in neuromodulation and neurological disorder management. Although traditional machine learning models, such as support vector machines and random forests, remain the most frequently used approaches, deep learning models, including convolutional neural networks, are increasingly being adopted, indicating a gradual shift toward more advanced analytical techniques.

## Discussion

### Principal Findings

This study conducted keyword analysis and topic modeling based on academic papers on smart bioelectronics published over the past 5 years. The analysis identified three major thematic areas: (1) bio-signal–based neuromodulation, (2) AI-driven neurological disease analysis, and (3) implantable bioelectronics and biomaterials. These findings demonstrate that smart bioelectronics are expanding across diverse domains, both technologically and clinically. In particular, they are emerging as a convergent technology that enables integrated disease management, spanning early detection, diagnosis, treatment, and continuous monitoring.

Furthermore, the coexistence and intermingling of terms such as “bioelectronic medicine” and “electronic drug” highlight the need for clearer conceptual definitions and a standardized classification system in this field. Establishing consistent terminology and conceptual frameworks will be essential to improve the efficiency, interoperability, and advancement of interdisciplinary research and technological applications.

From a technological perspective, conventional bioelectronic devices have traditionally relied on hardware-based electrical stimulation to induce therapeutic effects. In contrast, smart bioelectronics are increasingly developing into integrated systems that combine bio-signal sensing, wireless communication, and data-driven analytical capabilities. Consistent with these findings, the identified topics indicate a transition toward systems that combine continuous bio-signal monitoring, machine learning-based data analysis, and adaptive therapeutic functions.

These technological advancements are closely linked to their clinical relevance. Bio-signal–based approaches enable continuous and objective physiological monitoring, while AI-driven analytical methods support more accurate diagnosis and interpretation of complex neurological conditions. In addition, implantable bioelectronic systems facilitate real-time monitoring and closed-loop therapeutic interventions, allowing treatments to be dynamically adjusted based on patient-specific conditions. Collectively, these developments highlight the potential of smart bioelectronics to improve diagnostic accuracy, enhance treatment personalization, and support more efficient long-term disease management.

Despite these contributions, this study has several limitations. The dataset consisted of 92 papers, representing a moderate sample size for exploratory topic modeling. However, the relatively limited number of documents may affect the stability of probabilistic models such as LDA; therefore, the identified topics should be interpreted as indicative trends rather than definitive thematic structures. In addition, data collection was limited to the PubMed database, which may restrict the comprehensive coverage of the research landscape. Future research should expand the scope of literature collection to include multiple academic databases, such as IEEE Xplore and Embase, to improve coverage and robustness. The application of advanced natural language processing techniques, such as BERTopic, may further enhance analytical precision and enable more fine-grained topic extraction. These efforts will contribute to a more comprehensive understanding of the technological evolution and core research directions in smart bioelectronics. Smart bioelectronics represent a multidisciplinary research field encompassing medical technology, information technology, and life sciences. Continued integration of clinical research and technological innovation, along with collaboration among academia, industry, and health care systems, will be essential to accelerate the development of AI-driven medical data analysis systems and expand clinical applications, ultimately advancing next-generation precision health care.

### Conclusions

This study analyzed recent research trends in the field of smart bioelectronics by conducting keyword analysis and LDA-based topic modeling on 92 papers collected from PubMed. The findings reveal an increasing trend in research activity over the past 5 years, with topics evolving from bio-signal sensing and device-level technologies to AI-driven analysis, predictive modeling, and clinically oriented, adaptive bioelectronic systems for precision medicine. These results provide a foundation for shaping domestic research and development strategies in smart bioelectronics and offer insights for applications in digital health care, AI-driven therapeutics, and next-generation wearable medical devices. Despite these contributions, this study is limited by the relatively small number of papers analyzed and its reliance on a single database. Future research should integrate data from multiple international databases and adopt more advanced computational approaches, such as transformer-based topic modeling and network analysis, to better capture diverse research trends and uncover emerging subfields in bioelectronics.

## Supplementary material

10.2196/83092 Multimedia Appendix 1 Preliminary review of the paper.

## Abbreviations

AI: artificial intelligence
BoW: bag-of-words
LDA: latent Dirichlet allocation
TF-IDF: term frequency–inverse document frequency
