# Supplementary material for: Computational Insights Into Smart Bioelectronics in Digital Health Care (2020-2024): Topic Modeling Study
Source: JMIR Med Inform. 2026 Jun 23;14:e83092. doi: 10.2196/83092 (PMC13290107; doi:10.2196/83092)
Supplement: Multimedia Appendix 1 [file medinform-v14-e83092-s001.docx]

Appendix

Table. 1. Pre-review of the paper

| **Published** | **Journal** | **Keywords** | **Model/Methods** | **Ref** |
| --- | --- | --- | --- | --- |
| 2020 | Neuropsychopharmacology |  | PET metabolic analysis | 15 |
| 2020 | Neuroimage Clin | Adaptive deep brain stimulation, Brain-computer interface, Deep brain stimulation, Machine learning, Neural marker | Support Vector Machine (SVM), Random Forest | 16 |
| 2020 | Annu Int Conf IEEE Eng Med Biol Soc |  | Intracranial EEG (iEEG) | 17 |
| 2020 | IEEE J Biomed Health Inform | traumatic stress, physiological biomarkers, vagal nerve stimulation, wearable bioelectronic medicine, wearable neuromodulation, wearable sensing | Support Vector Machine (SVM), Random Forest | 18 |
| 2020 | Annu Int Conf IEEE Eng Med Biol Soc |  | Subthalamic LFP signals | 19 |
| 2020 | Nat Commun |  | sensor | 20 |
| 2020 | Brain Stimul | Closed-loop stimulation, Mental stress, Noninvasive stimulation, Physiological biomarkers, Transcutaneous cervical stimulation, Traumatic stress, Vagal nerve stimulation, Wearable bioelectronic medicine | Closed-loop | 21 |
| 2020 | Nat Commun |  | machine learning / deep learning | 22 |
| 2020 | JMIR Mhealth Uhealth | bioelectronic medicine, biomarker, cardiovascular, digital biomarkers, dynamic models, neuromodulation, noninvasive, state space, vagus nerve stimulation, wearable sensing | state-space modeling | 23 |
| 2020 | Sensors (Basel) | PEDOT:PSS, bioelectronic device, electrocardiography (ECG), gelatin, hydrogel electrode | hydrogel + electrode | 24 |
| 2020 | IEEE Trans Biomed Eng | Soft bioelectronics, stretchable electrodes, flexible hybrid electronics, blepharospasm, electrophysiology, quantitative diagnostics | CNN | 25 |
| 2020 | Proc Natl Acad Sci U S A | bioelectronics, cerebral hemodynamics, near-infrared spectroscopy, wearable electronics |  | 26 |
| 2021 | Nat Commun |  | machine learning | 27 |
| 2021 | Neuroimage Clin | Anterior limb of the internal capsule, Deep brain stimulation, Machine learning, Nucleus accumbens, Obsessive–compulsive disorder, Treatment outcome prediction | correlation analysis | 28 |
| 2021 | Mov Disord | Parkinson's disease, cognition, deep brain stimulation, machine learning, prediction, quantitative EEG, subthalamic nucleus | Support Vector Machine (SVM), Random Forest | 29 |
| 2021 | Brain Stimul | Amygdala stimulation, Feature learning, Hippocampus, Interpretable machine learning, Local field potential, Memory, Neurophysiological biomarkers | Random Forest + feature importance | 30 |
| 2021 | Transl Psychiatry |  | Support Vector Machine (SVM), Random Forest | 31 |
| 2021 | Nat Commun |  | Support Vector Machine (SVM), Random Forest | 32 |
| 2021 | Clin Neurophysiol | Cognition, Deep Brain Stimulation, Machine learning, Parkinson’s Disease, Quantitative EEG | Support Vector Machine (SVM), Random Forest | 33 |
| 2021 | JMIR Mhealth Uhealth | electrodermal activity, health monitoring, machine learning, pain assessment, post-op patients, recognition, wearable electronics | signal analysis | 34 |
| 2021 | PLoS One |  | CNN | 35 |
| 2021 | Contrast Media Mol Imaging |  | CNN | 36 |
| 2021 | Sensors (Basel) | cognitive workload, microwave brain stimulation, physiological biomarker, wearable bioelectronic medicine | wearable sensing | 37 |

| 2021 | Adv Mater | carbon nanotubes, machine learning, motion artifacts, personalized healthcare, pulse wave monitoring, smart textiles | sensor + machine learning | 38 |
| --- | --- | --- | --- | --- |
| 2021 | J Neural Eng | bioelectronics, cortical stimulation, neuroengineering, noninvasive brain stimulation, photovoltaics, whisker cortical areas, wireless brain stimulation |  | 39 |

| 2021 | J R Soc Interface | bioelectronic devices, feedback control, wound healing | bioelectronic device | 40 |
| --- | --- | --- | --- | --- |
| 2021 | Biosens Bioelectron | Bioelectronic sensors, Multiplexed detection, Personalized nutrition, Saliva monitoring, Vitamin C, Vitamin D | dual bioelectronic sensor chip | 41 |
| 2021 | Biosens Bioelectron | Bacteriophage, Bioelectronic nose, Genetic engineering, Lung cancer, Surface chemistry | deep learning | 42 |
| 2022 | AMIA Annu Symp Proc |  | machine learning | 43 |
| 2022 | Sci Rep |  | deep learning vision models | 44 |
| 2022 | Cereb Cortex | deep brain stimulation, functional connectivity, magnetoencephalography, parkinson’s disease, synchrony | neural signal analysis and network connectivity analysis | 45 |
| 2022 | Neurotherapeutics | Brain connectivity, Deep brain stimulation, Parkinson’s disease, Prediction, Subthalamic nuclei | statistical correlation | 46 |
| 2022 | Brain Imaging Behav | Human, Interoception, Machine learning classification, Obesity, Reward, Saliency, fALFF, rs- fMRI, tVNS | Support Vector Machine (SVM), Random Forest | 47 |
| 2022 | Neurosurgery |  | multivariate statistical analysis | 48 |
| 2022 | Stereotact Funct Neurosurg | Clinical prediction models, Deep brain stimulation, Machine learning, Motor response, Outcome prediction, Parkinson’s disease | Random Forest, gradient boosting models | 49 |
| 2022 | CNS Neurosci Ther | brain morphology, efficacy, machine learning, subthalamic nucleus deep brain stimulation | Support Vector Machine (SVM), Random Forest | 50 |
| 2022 | Sci Rep |  | Support Vector Machine (SVM), Random Forest | 51 |
| 2022 | Int J Numer Method Biomed Eng | DBS efficacy, Parkinson's disease, deep learning, magnetic resonance imaging, neuroprotection | CNN-based models | 52 |
| 2022 | CNS Neurosci Ther | drug-resistant epilepsy, machine learning, scalp electroencephalography, synchronization, vagus nerve stimulation | Support Vector Machine (SVM), Random Forest | 53 |
| 2022 | Brain | Parkinson’s disease, deep brain recording, hidden Markov modelling, machine learning, time-series analysis | spectral analysis | 54 |
| 2022 | Sensors (Basel) | ANT-DBS, LFPs, closed-loop stimulation, epilepsy, target localization | LFP signal analysis | 55 |
| 2022 | Adv Sci (Weinh) | amino acid engineering, energy harvesting technology, intrinsic crosslink, mechanical property, silk fibroin, wearable bioelectronics |  | 56 |
| 2022 | Front Endocrinol (Lausanne) | artificial pancreas, biosensor, closed-loop simulation, insulin therapy, micro-electrode array, pancreatic islets, type 1 diabetes | islet-based biosensor + control algorithm | 57 |
| 2022 | Science |  | closed-loop | 58 |
| 2022 | Sci Adv |  | AI-based binary classification | 59 |
| 2022 | Am J Case Rep | Amputation, Residual Limb Pain, Analgesia, Electromagnetic Fields, Phantom Limb, Pulsed Radiofrequency Treatment, Radiofrequency Therapy | wearable device | 60 |
| 2022 | Sensors (Basel) | 3D printing, COVID-19, Internet of Things, Medicine 4.0, artificial intelligence, bioelectronics, edge computing, healthcare, medical device, safety, wearables | machine learning | 61 |
| 2022 | Small | biocompatible inks, drawn-on-skin, electrophysiology, wearable bioelectronics |  | 62 |
| 2022 | Nano Lett | bioelectronics, cardiac organoid, liquid metal, multimodal characterization, pressure sensor |  | 63 |
| 2022 | Biomaterials | Computational modelling, Foreign body reaction, Intraneural stimulation, Nerve histology, Neural interface, Peripheral nervous system |  | 64 |
| 2022 | Sensors (Basel) | bioelectronic implants, capacitive sensing, computing in medical devices, implant technology, instrumented medical device, smart implants | capacitive sensor network | 65 |
| 2023 | Nature |  | computational signal analysis and statistical modeling | 66 |
| 2023 | J Neuroeng Rehabil | AI, Automatic calibration, Electrical stimulation, Neuropathy, Neurostimulation, Reinforcement learning, Sensory feedback, TENS | reinforcement learning | 67 |
| 2023 | CNS Neurosci Ther | brain functional connectivity, ictal EEG recording, scalp EEG, support vector machine, vagus nerve stimulation | machine learning classification models | 68 |
| 2023 | Brain Stimul | Closed-loop, Memory, Neuromodulation, Stimulation, TBI | neural signal analysis | 69 |
| 2023 | Cereb Cortex | brain–computer interface, episodic memory, machine learning, neuromodulation | Support Vector Machine (SVM), Random Forest | 70 |
| 2023 | Neuroimage Clin | Deep brain stimulation, Machine learning, Parkinson's disease, Resting-state functional magnetic resonance imaging |  | 71 |
| 2023 | Sci Rep |  |  | 72 |
| 2023 | Biomed Eng Online | Computer vision, Dementia, Gait analysis, Machine learning, Parkinson’s disease | CNN-based vision models | 73 |
| 2023 | BMC Neurol | EEG, Machine learning, Parkinson’s disease, Tremor | Support Vector Machine (SVM), Random Forest | 74 |
| 2023 | Adv Sci (Weinh) | bioelectronic sensors, flexible electronics, transparent devices |  | 75 |
| 2023 | Neurogastroenterol Motil | bioelectronics, diagnostics, functional gastrointestinal disorders, gastric motility |  | 76 |
| 2023 | Proc Natl Acad Sci U S A | bioelectronics, virtual reality, wearable | closed-loop | 77 |
| 2023 | ACS Sens | OECT, PEDOT:PSS, bioelectronics, non-enzymatic, organic electrochemical transistor, sensor, smart dressing, textile, uric acid sensing, wearable, wound healing | organic electrochemical transistor(OECT) textile sensor | 78 |
| 2023 | Brain Stimul | Amputation, Bionic prosthetics, Electroceuticals, Embodiment, Neuromodulation, Neuropathic pain, Neuroprosthetics, Neurostimulation, Peripheral nerve stimulation, Phantom limb, Phantom limb pain, Sensory restoration, Transcutaneous electrical nerve stimulation |  | 79 |
| 2023 | Appl Psychophysiol Biofeedback | Brain entrainment, Insomnia, Sleep, Sleep treatment |  | 80 |
| 2023 | Pain Pract | ambulatory surgery, analgesia, outpatient surgery, postoperative analgesia | wearable device | 81 |
| 2023 | Adv Sci (Weinh) | Parkinson's disease, finger flexibility, hand muscle strength, hand stability, smart glove, wearable bioelectronics | machine learning | 82 |
| 2023 | Small Methods | MXenes, bioelectronics, high-density surface electromyography, human-machine interfaces, rehabilitation | machine learning | 83 |
| 2023 | Nat Commun |  | machine learning | 84 |
| 2023 | Adv Healthc Mater | bioelectronics, biological circuit modelling, electrochemical impedance spectroscopy, electrodes, tissue engineering |  | 85 |
| 2023 | Cell | cardiomyocyte, electrophysiology, flexible bioelectronics, human-induced pluripotent stem cell, in situ RNA sequencing, multimodal analysis, neuron, spatial transcriptomics, stretchable electronics |  | 86 |
| 2024 | Neurotherapeutics | Deep brain stimulation, Deep learning, Multi-instance, Parkinson's disease, Prediction model | a multimodal deep learning framework combined with multi-instance learning (MIL) | 87 |
| 2024 | Asian J Psychiatr | Functional connectivity, Machine learning, Major depressive disorder, Neuroimaging biomarker, Transcutaneous auricular vagus nerve stimulation | machine learning | 88 |
| 2024 | Laryngoscope | deep learning, obstructive sleep apnea, upper airway stimulation | deep learning–based predictive modeling(Deep Neural Network (DNN)) | 89 |
| 2024 | Int J Comput Assist Radiol Surg | Deep Q-learning, Deep brain stimulation, Optimization, Path planning, Reinforcement learning | reinforcement learning agents (Q-learning / Deep Q-Network (DQN)) | 90 |
| 2024 | J Neural Eng | Shannon equation, machine learning, neuromodulation, safe stimulation | Random Forest, Gradient Boosting, Neural Networks | 91 |
| 2024 | Nat Commun |  | computational modeling of neural activation and optimization algorithms | 92 |
| 2024 | IEEE J Transl Eng Health Med | Deep brain stimulation optimization, Parkinson’s disease, deep learning, fMRI, unsupervised feature extraction. | CNN-based deep learning models | 93 |
| 2024 | Sci Rep | Coil placement, Deep learning, Electric field optimization, Multi-task learning, Transcranial magnetic stimulation | 3D CNN | 94 |
| 2024 | Neural Netw | Attention, Classification, Deep brain stimulation, Microelectrode recording, Residual neural network, Temporal domain | Residual Neural Network (ResNet) | 95 |
| 2024 | Neurol Sci | ChatGPT, Deep Brain Simulation, Parkinson’s disease | GPT-3.5 | 96 |
| 2024 | Clin Neurophysiol | Classification, MEG, Machine learning, Parkinson’s disease, Subthalamic nucleus, Tremor, Voluntary movements | Signal feature extraction | 97 |
| 2024 | J Neurosci | cortical thickness, neuroanatomy, neurodevelopment, neuromodulation, surface area, transcranial direct current stimulation | Statistical regression models | 98 |
| 2024 | Adv Sci (Weinh) | ChatGPT-bioelectronic interface, electrochemical multimodal device, multimodal biosensing, paper based, personal healthcare | ChatGPT-based AI | 99 |
| 2024 | Adv Sci (Weinh) | DNA hydrogel, OECT, bioelectronics, electrical stimulation, wound management | closed-loop | 100 |
| 2024 | Adv Sci (Weinh) | bioelectronics, cardiomyocytes, electrophysiology, multi‐electrode arrays, optogenetics |  | 101 |
| 2024 | Nat Commun |  | Sensor | 102 |
| 2024 | Biosens Bioelectron | Direct electron transfer, Implantable bioelectronics, Lactate sensor, Near field communication, Periprosthetic joint infection | a wireless implantable electrochemical sensing platform | 103 |
| 2024 | Proc Natl Acad Sci U S A | closed-loop management, in situ therapy, remote care, temperature spatial mapping, wound inflammation |  | 104 |
| 2024 | PLoS One |  |  | 105 |
| 2024 | Biosens Bioelectron | Electrical stimulator-based pacing, Field potential monitoring, Impedimetric contraction strength monitoring, Large-area microelectrode array, Rotor pattern detection |  | 106 |
